# Advances in pathogenesis and treatment of ocular involvement in Behcet’s disease

**DOI:** 10.3389/fimmu.2023.1206959

**Published:** 2023-09-29

**Authors:** Suibin Lin, Zhirong Xu, Zhiming Lin, Baozhao Xie, Junmei Feng

**Affiliations:** ^1^ Department of Gynaecology and Obstetrics, Zhangpu Hospital, Zhangzhou, China; ^2^ Department of Internal Medicine, Zhangpu Hospital, Zhangzhou, China; ^3^ Department of Rheumatology and Immunology, Third Affiliated Hospital of Sun Yat-Sen University, Guangzhou, China; ^4^ Department of Rheumatology and Immunology, the Seventh Affiliated Hospital of Guangxi Medical University (Wuzhou Gongren Hospital), Wuzhou, China; ^5^ Department of Rheumatology and Immunology, Sun Yat-Sen Memorial Hospital of Sun Yat-Sen University, Guangzhou, China; ^6^ Department of Rheumatology and Immunology, Nanfang Hospital of Southern Medical University, Guangzhou, China

**Keywords:** Behcet’s disease, ocular involvement, Behcet’s uveitis, immunopathogenesis, immunogenetic, biological agent, pregnancy

## Abstract

Behcet’s disease (BD) is a chronic multi-systemic disease characterized by relapsing-remitting oral ulcers, genital ulcers, ocular inflammatory involvements, and numerous other systemic features. Ocular involvements are quite common in BD and may cause severe tissue damage and potentially blindness. Even though the pathogenesis of BD remains ambiguous, growing evidences have shown that genetic factors, environmental triggers and immunological abnormalities play significant roles in its development and progression. Novel biotherapies targeting IFN-γ, TNF-α and interleukins have been used in recent years. In this review, we mainly pay attention to the ocular involvement of BD, and discuss the current understanding of mechanisms and advances in therapeutic approaches, especially novel biologics. Finally, we discuss the management in patients with pregnancy.

## 1 Introduction

Behcet’s disease (BD) is a chronic multi-systemic disease pictured by relapsing-remitting oral aphthous ulcers, genital ulcers, ocular inflammatory involvements, skin impairment, and numerous other symptoms (1). BD is more prevalent in countries along the ancient Silk Road, from East Asian countries to Mediterranean basin (2). Turkey was reported to have the highest prevalence rate around the world with up to 420 cases in every 100,000 residents (3). Ocular involvement is most frequently observed in BD, with a prevalence of 40 to 70% in patients with BD (3, 4). In addition, uveitis is the most common and most classic ocular manifestation, which occurs in 60 to 80% of cases (5). It is usually acute and recurrent, and presents non-granulomatous panuveitis related to retinal vasculitis (5). It may cause severe tissue damage in the eyes and potentially lead to blindness. Other ocular manifestations are relatively rare, such as episcleritis, scleritis, conjunctival ulcers, keratitis, orbital inflammatory disease, isolated optic neuritis and oculomotor palsies (5).

Even though the pathogenesis of BD remains obscure, recent studies have indicated that both genetic and environmental factors play an important part in its development and progression. Due to the advances of recent genome analysis research, a number of susceptibility genes related to the pathophysiology of non-infectious uveitis in BD have been discovered (2). Immunological aberrations in both innate and acquired immune systems are found to be implicated in the mechanisms of BD.

Treatment of ocular involvement in BD includes corticosteroids and immunosuppressant therapy. Conventional immunosuppressors such as azathioprine and cyclosporine A were often used, and they were recommended by EULAR in 2018 (6). However, potential side effects have limited their application and require close monitoring. For example, azathioprine showed hematologic, hepatic and neurotoxicity, whereas cyclosporine A presented nephrotoxicity and hypertension (5). Apart from conventional immunosuppressors, novel biologic agents such as interferon-a (IFN-α), tumor necrosis factor-a (TNF-α) inhibitors, different bio-agents targeting interleukins and related receptors were found to show a significant improvement in the visual prognosis of BD patients.

The main target of this review is to discuss latest advances in areas regarding pathogenesis of BD ocular involvement and related novel treatment approaches.

## 2 Pathogenesis

### 2.1 Genetic susceptibilities

Various evidences have shown that host genetic factor play a crucial role in the pathogenesis of BD. Familial aggregation and high recurrence risk in siblings suggested the involvement of genetic factor in BD development (7, 8). *HLA-B51* has been considered as the strongest predisposing gene in BD. Recent years, with the development of novel research techniques such as genomewide association study (GWAS), more and more genes are identified to be associated with pathogenesis of BD ocular involvement (2).

#### 2.1.1 HLA genes

It has been first reported by Ohno et al. in 1973 that HL-A5 (later known as HLA-B5), which includes HLA-B51, has a strong association with BD among the Japanese population (9). After this initial genetic discovery, *HLA-B51* was then found to have a strong association with BD in 1982 (10). Afterwards, this association was further confirmed in multiple studies among different populations of BD patients (11–22). *HLAB51* allele accounts for about 19% of the genetic susceptibility and is by far the most powerful genetic factor in BD (23, 24). A meta-analysis of 78 independent researches indicated that individuals with *HLA B51/B5* are 5.78 times more likely to develop BD compared with non-carriers (25). The strong relationship between *HLA-B51* and BD was also confirmed in two large GWAS studies recently, which was conducted in Turkish and Japanese populations respectively (26, 27). In addition, several studies have reported that *HLA-B51* positive BD patients have a higher risk of eye complications and genital ulcers in comparison with *HLA-B51* negative BD patients, indicating a potential prognostic valve of *HLA-B51 (* 28, 29).

Apart from *HLA-B51*, other HLA genes also showed disease susceptibility to BD. *HLA-A26* was identified to be correlated with BD in various populations such as Chinese Taiwanese, Greek, Japanese, South Korean, Saudi Arabian and Turks (30–35). Carriers of this allele in Japanese and South Korean BD patients were observed with a higher risk of ocular involvement and a poor visual prognosis (36–38). Besides, the *HLA-A02, HLA-A24, HLA-A26, HLA-A31, HLA-B27* and *HLA-B57* were recognized as risk alleles, and the *HLA-A03, HLA-B15, HLA-B35, HLA-B49* and *HLA-B58* were recognized as protective alleles to BD development in different populations (30, 39–46).

#### 2.1.2 HLA related genes

The endoplasmic reticulum aminopeptidase-1 (*ERAP1*) gene, locating at 5q15, encodes an enzyme that trim the proteasome-processed antigenic peptides for attachment to MHC-I molecules and expression on cell surface (47, 48). Genome-wide association tests were performed in the subgroup of GWAS discovery collections with BD uveitis and two SNPs in *ERAP1* were identified to confer risk for BD uveitis recessively—rs10050860 and rs17482078, which encode p.Asp575Asn and p.Arg725Gln alterations in *ERAP1* respectively (49). And the association was further confirmed by a meta-analysis of the p.Arg725Gln SNP, which combines discovery and replication collections of Turkish population including 790 BD uveitis and 1879 controls (49). In addition, several studies have shown that the *ERAP1* variants present higher susceptibility for BD in *HLA-B51* positive cases (44). Homozygosity of *ERAP1* p.Arg725Gln was more likely to develop BD in *HLA-B51* positive individuals than in *HLA-B51* negative cases (OR=3.78, 95% CI=1.94-7.35 vs OR=1.48, 95% CI=0.78-2.80) (44). It is assumed that variants of *ERAP1* might influence the repertoire of disease-associated peptides that attach to HLA-B51 protein and contributes to disease susceptibility.

MHC class I chain-related gene A (*MICA*) is positioned in the centromeric region of *HLA-B*. MICA proteins present homology with classical HLA molecules and act as ligands for NKG2D receptors, which are expressed on natural killer-cell, γδT cells and αβCD8T cells (50). Studies have shown that MICA*009, MICA*019 and MICA*A6 alleles are associated with BD susceptibility (22, 51, 52). A strong linkage disequilibrium between MICA*A6 and *HLA-B51* was observed by a Korean study in both BD cases and healthy controls (53). However, independent association between *MICA* and BD was not observed in recent GWAS studies (26, 27, 41).

#### 2.1.3 Non-MHC region genes

*IL-10* gene is positioned at 1q21-32. IL-10 is a cytokine with anti-inflammation function that impedes antigen presentation by reducing the MHC expression on cellular surfaces and hinders the co-stimulation activity of macrophages, T lymphocytes and NK-cell activation (54). Existing genotype analyses reported different *IL-10* genotypes associated with BD (55, 56). More recently, GWAS have identified numerous variants within the *IL-10* gene related to BD in various ethnic populations, including rs1800872, rs1518111, rs1554286, and rs1800871 (26, 27, 55, 57, 58). In addition, the rs1518111 and rs1800871 were also found in non-infectious uveitis patients (26, 27, 59). As shown in expression studies, the genetic *IL-10* variants were related to decreased levels of IL-10, which may induce a susceptible inflammatory state and increase BD susceptibility (26). These results indicated that *IL-10* gene polymorphisms may contribute to the development of ocular involvement in BD.

Pro-inflammatory cytokines such as IL-12 and IL-23 promote Th1 differentiation and stimulate Th17 proliferation, respectively. GWAS and meta-analysis reported several SNPs in *IL23R-IL12RB2* and *IL12A* related to BD susceptibility in different populations (26, 27). Furthermore, a strong correlation between *IL-23R* rs17375018 and BD uveitis was found in a Chinese Han population (60). This study also reported that *IL-23R* SNP rs11209032 was associated with uveitis susceptibility in patients with BD.

Due to the increasing GWAS researches and meta-analyses in recent years, more and more SNPs were identified to be linked to BD uveitis, including SNPs of *UBAC2, STAT4, TNFAIP3, CCR1, KLRC4, IL1A-IL1B, IRF8, CEBPB-PTPN1, ADO-EGR2, RIPK2, LACC1, JRKL-CNTN5, FUT2, MEFV*, *NCOA5* genes and so on (2, 61).

### 2.2 Environmental triggers

It is not sufficient to elucidate the development of BD by genetic abnormalities alone. In patients with genetic susceptibility to BD, environmental triggers have long been proposed. The environmental triggers contain infections caused by bacteria and viruses, as well as abnormal autoantigens. Bacterial species like Streptococcus sanguis, Helicobacter pylori, and Mycoplasma and many Herpesviridiae such as HSV1, CMV, VZV and EBV have been investigated (62). Furthermore, Streptococcus sanguis and HSV have been proved to induce BD-like symptoms in mice models respectively (63, 64). Autoantigens are suggested to play a crucial role in the mechanisms of BD through molecular mimicry (1). Researchers have identified several autoantigens in BD ocular involvement, such as the heat-shock protein 60 kDa (HSP60), retinal S antigen and interphotoreceptor retinoid-binding protein (IRBP) (62).

Human HSP60 and counterpart HSP65 related to S. sanguinis were revealed to have high homology. Four peptides of bacterial HSP65 (111-125, 154-172, 219-233, and 311-326) were found to stimulate lymphoproliferative immune reactions and these peptides showed 50%-80% homology to the corresponding human HSP60 (65, 66). It is suggested that cross-reaction between human HSP60 and bacterial HSP65 may induce autoimmune disorders and play a significant part in the pathogenesis of BD. Furthermore, several animal studies have shown that immunization of HSP peptide to rats by different routes (subcutaneously, orally or nasally) could induce clinical and/or histological uveitis (67, 68). It is also shown that antigenic homology exists between HSP60 and retinal antigen (69). Thus, HSP is believed to participate in the ocular lesions of BD.

Retinal S-antigen is a well-studied autoantigen for autoimmune uveitis. It can induce experimental autoimmune uveitis (EAU) in animal models, which is similar to human uveitis both clinically and histologically. Besides, it is suggested that administration with human S-Antigen-Ig fusion protein to rats is capable of preventing EAU. On the other hand, autoimmunity to S-antigen has been confirmed in BD patients with retinal vasculitis and uveitis (70, 71). It is also reported that some epitopes of S-antigen are homologous with certain amino acid regions of HLA-B51 and HLA-B27 (72). These findings indicated that retinal S-antigen may be involved in the mechanisms of BD ocular manifestations.

IRBP is considered to facilitate transport of retinoids between the retina and pigment epithelium (73, 74). IRBP has 1264 amino acid residues and consists of four repeating units, with one uveitogenic site in each unit. In particular, strong uveitogenicity is elicited in the fourth repeated unit. IRBP and derived peptides are widely used to induce EAU, especially in mice (75). Injection of bovine IRBP in Lewis rats induced severe autoimmune uveoretinitis (76, 77). And different doses of antigen and the categories of animals applied could lead to different spectrum of uveoretinitis, from hyperacute to chronic. Furthermore, several studies have demonstrated immune responses to IRBP in BD patients with uveitis (70). Cellular autoimmunity and cytokine changes were recognized in BD patients with uveitis, with significantly higher titers of IL-6, IL-17, and IFN-γ compared to healthy controls. And BD patients with active uveitis identified higher IFN-γ compared to remissive uveitis. Therefore, IRBP is potential participated in the development of BD uveitis.

### 2.3 Immunological aberrations

Immune system dysfunction has been observed in BD. T cells are recognized as major lymphocyte subsets implicated in BD development. Growing evidences have shown that NKT cells, γδT cells, Th cells, Treg cells and cytokines with different functions may play significant roles in the pathogenesis of BD ocular involvement and might be potential targets for treatment.

NK cells are main components of the innate immunity. Their functions contain both nonspecific cytotoxic activity and regulatory roles in the innate and acquired immune responses by cytokine production (78). However, inconsistent results have been indicated in BD patients. Some studies have reported that NK cells activity was increased in active BD patients. Inversely, decreased levels of CD56brightCD16- and CD56dimCD16+ NK cells in peripheral blood were reported in patients with BD recently (79). In addition, a study found that the level of CD8+CD56+ NKT cells was higher in aqueous humor as well as peripheral blood in BD patients with uveitis (80). Further study revealed that CD8brightCD56+ T cells in Behcet’s uveitis possess functional NK receptors and display strong cytotoxic effect via Fas-ligand dependent pathway and perforin-dependent pathway (81). Both the clinical severity and histopathological severity of uveoretinitis were significantly milder in experimental autoimmune uveoretinitis-susceptible mice depleted of NK cells (81). Based on the secreted cytokines, NK cells is classified into different subtypes, such as NK1, NK2, NK22, NK17 and NKreg cells. It is indicated that the NK1/NK2 paradigm can regulate pathogenic Th1 or Th2 biased immune reactions in particular. NK2 cells were found to have a significant association with BD in remission stage (62). NK2 cells could inhibit INFγ secretion by Th1 cells *in vitro (* 82). This study suggested that NK cells might control disease remission in patients with BD through modulating Th1 response which is mediated by NK2 cells (82). The characteristic of NK cells and NK1/NK2 paradigm requires further investigation especially in BD patients with ocular manifestations.

It is well known that γδ T cells are involved in regulating autoimmune response. In patients with BD, researchers have found that γδT cells were enriched, activated, and highly proliferative in response to various microbial infections (83–85). It is also found that γδT cells associate with active stage of BD, as well as elevated CD69 expression and increased IFN-γ and TNF-α production (1). The Vγ9/Vδ2T cells are the major subsets of γδT cells which represents up to 90% of the circulating γδT cells in humans. TCR Vγ9Vδ2+ Th1-like cells can be generated from intra-ocular fluid in BD uveitis patients (86, 87). Moreover, activated γδT cells exacerbate EAU by promoting the activation of IL-17+ uveitogenic αβT cells (88). These finding indicated that γδT cells may contribute to the pathogenesis of uveitis in BD.

**Figure 1:**
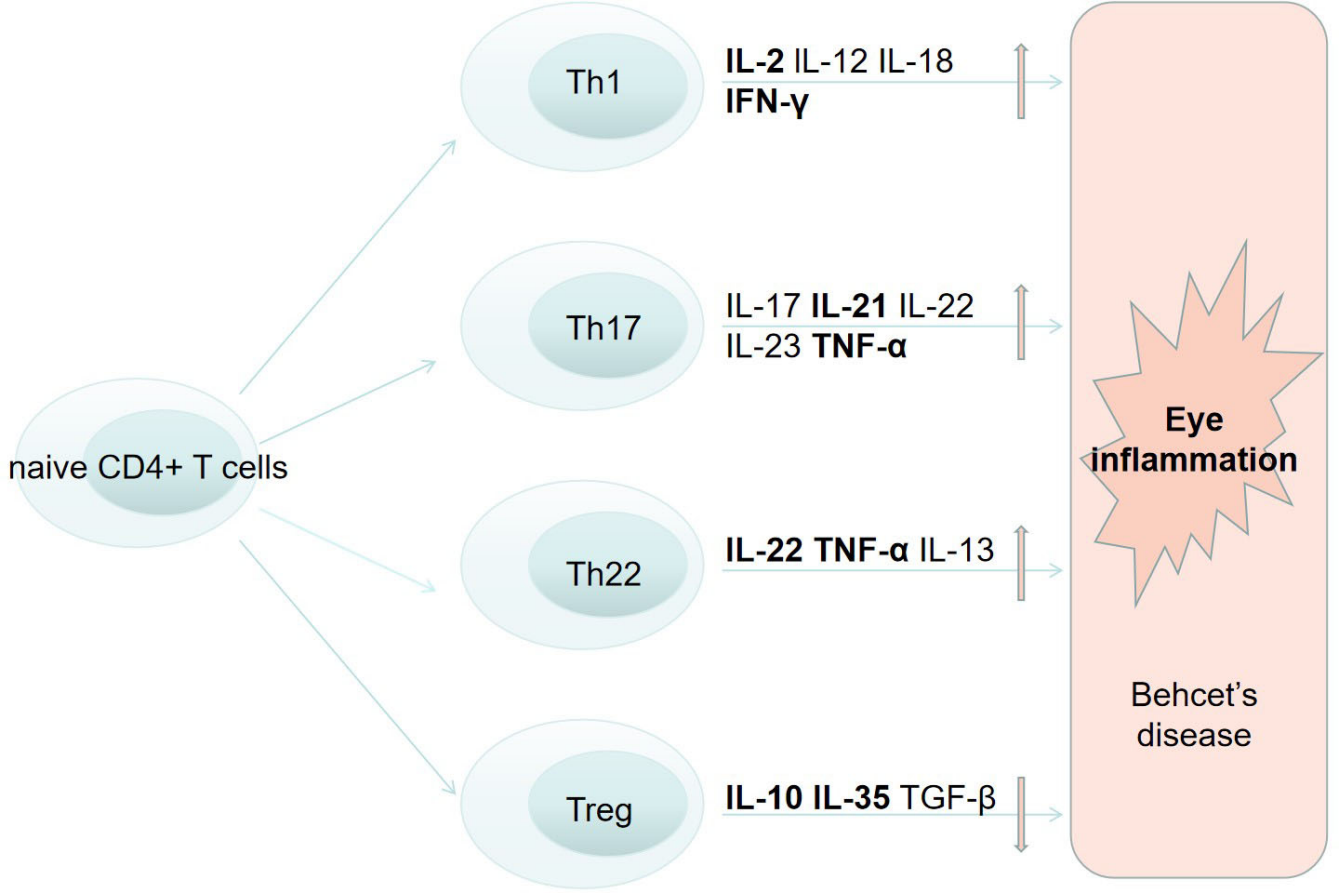
The role of T cells in the pathogenesis of Behcets disease and eye inflammation.

Elevated levels of Th1 cells and related cytokine production (including IL-2, IL-12, IL-18, and IFN-γ) was found in active BD patients (89, 90) (**Figure 1**). Different studies also observed higher IFN-γ in aqueous humor of BD patients than that of other diseases (90, 91).

Th17 cells can regulate inflammation and autoimmunity by producing different cytokines, for example IL-17A, IL-17F, IL-21, IL-22, and IL-23 (92). According to previous investigations, higher expression of Th17 cells and relevant cytokines were found in active BD patients rather than inactive patients, as well as in the same patient of remission stage (93–95). IL-23, in cooperation with IL-6, is able to facilitate the differentiation, survival and maintenance of Th17 cells. By inducing pro-inflammatory cytokines production, IL-23 is capable of enhancing Th17 cell immune response (96). The IL-17/23 axis is believed to play a significant part in mediating inflammatory responses in BD (1). In addition, IL21 can stimulate Th17 differentiation and modulate Th1, Tregs and B cells (97). High titers of IL-21 were observed in PBMC from individuals with active BD. IL-21 and IL-2, produced by T lymphocytes in the retina, are also found to be associated with development of EAU (98). These findings suggests that Th17 pathway may be correlated with BD uveitis (**Figure 1**).

Th22 cells are differentiated from activated naive CD4+ T cells in the presence of IL-6 and TNF-α. Th22 cells not only secrete pro-inflammatory cytokines like IL-22 and TNF-α, but also express chemokine receptors such as CCR4, CCR6, and CCR10 (99). Th22-type T lymphocyte clones were constructed by researchers from ocular samples of active uveitis in BD patients (100). These clones secreted a great number of IL-22 and TNF-α (**Figure 1**). With the treatment of anti-TNF-α and anti-IL-6 agents, theses clones failed to produce IL-22. Moreover, large quantities of IL-22 were produced by intraocular T cells from EAU animal models of BD with retinal antigens. Higher IL-22 was also found in the supernatant of stimulated PBMCs in active Behcet’s uveitis patients compared to BD patients without eye involvement or in healthy controls (101). And the association between IL-22 level and severity of retinal vasculitis and anterior chamber inflammation was also reported.

Treg cells are able to produce immunosuppressive cytokines including IL-10, IL-35, and TGF-β and are vital in regulating immune responses (**Figure 1**). However, conflicting results of Treg cells levels were found in different studies with BD. Some studies found increased Tregs in peripheral blood and CSF (102), while others found decreased Tregs (97). Recent studies have reported abnormal expression of IL-35 in EAU, functional analysis suggested that IL-35 play an important part in the onset and development of EAU as well (103).

Proinflammatory cytokines in BD patients mainly include IL-1, IL-6, and TNF-α and are increased in BD patients (104). These inflammatory cytokines have also been observed in ocular tissue of BD patients and inflammation was induced in animal models after intraocular injection (104). Accumulating findings suggested that these cytokines are major inflammatory mediators participated in the development BD and may be promising targets for treatment.

Anti-inflammatory cytokines in BD mainly include IL-37, IL-27 and IL-10. Recent studies found decreased IL-37 levels in BD patients, and functional analysis suggested a negative correlation between IL-37 expression and BD development (105, 106). IL-27 is able to inhibit EAU by inhibiting Th17 cells proliferation and inducing the production of IL-10 (107–109). And the expression level of IL-27 was lower in active BD cases in comparison with controls. Decreased levels of anti-inflammatory cytokines are associated with BD development and their association with BD ocular involvement needs further investigation.

## 3 Novel treatment

Traditional treatment measures of BD ocular involvement include topical or systemic administration of glucocorticoid and conventional immunosuppressors such as Azathioprine (AZA), Cyclosporine A (CYC) and Tacrolimus. With the occurrence of emerging biological agents, visual outcomes and prognoses of patients have greatly improved during the past few decades.

### 3.1 IFN-α-2a

IFN-α was the first biological agent applied to treat BD due to its anti-viral function against HSV 1 (110). *In vitro* studies have indicated that IFN-α can lower peripheral γδT cells and impede T cell attachment to endothelial cells (111, 112). IFN-α-2a, as well as azathioprine, cyclosporine-A and monoclonal anti-TNF antibodies,has been considered as choices for BD patients with inflammatory eye disease affecting the posterior segment according to EULAR recommendations in 2018 (6). Moreover, treatment with high-dose glucocorticoids, infliximab or IFN-α were also recommended in patients with an acute sight-threatening uveitis (6). Several retrospective and prospective studies have shown that IFN-α-2a presented effectiveness in severe Behcet’s uveitis or uveitis refractory to conventional immunosuppressive medications (113–125). Treatment of IFN-α-2a can improve or stabilize visual acuity and achieve long-lasting remission of BD, even in some patients discontinued treatment (125–127).

### 3.2 TNF-α antagonists

TNF-α antagonists can be classified as monoclonal antibody and soluble receptor. Infliximab, adalimumab, and golimumab belong to the former group whereas etanercept belongs to the latter. Anti-TNF-α therapy possess a fast curative effect on all the clinical symptoms of BD and have also been widely used in severe or refractory Behcet’s uveitis (128–130). Rapid remission of uveitis could be gained after one day while benefit for visual acuity could be achieved within one week. Infliximab and adalimumab are regarded as first-line immunosuppressive agents to treat ocular diseases in BD. In patients who failed treatment using a first TNF-α inhibitor, switching to a different anti-TNF-α biologic should be taken into consideration.

Patients with short disease duration (<18 months) showed better visual effect when treated with infliximab, possibly due to decreased background vascular leakage before permanent eye injury occurs (131). Earlier initiation of infliximab may improve the outcome of Behcet’s uveitis (132). Short-term efficacy of infliximab in Behcet’s uveitis treatment has been well elucidated in many studies (133, 134). However, several factors are limiting the use of infliximab, including its high costs, side effects and lack of efficacy. A discontinuation of infliximab was reported in 15% patients in a recent study (135).

Adalimumab is also recommended in severe, intractable BD uveitis, including patients who are resistant to infliximab treatment, and has shown significant benefit in vision, reduction use of corticosteroid and immunosuppressors, and long-term remission (136). A retrospective study found equivalent efficacy of infliximab versus adalimumab (with response rate of 95-97%) in treatment of BD uveitis, and the rates of complete response or event-free survival showed no significant difference (137). A multicenter retrospective observational study was conducted in Behcet’s uveitis patients to evaluate the efficacy and safety of adalimumab (138). This study found significantly reduction of ocular inflammatory flares at 12-months follow-up, as well as significant improvement of best corrected visual acuity, and reduction of macular thickness and vasculitis occurrence during follow up. Randomized clinical trials towards adalimumab were conducted in non-infectious uveitis including BD patients with inadequate control by steroids (VISUAL I, VISUAL II, and VISUAL III), and the efficacy of adalimumab was observed with a reduced risk of optical activity and visual damage (139–141).

Golimumab appears to have potent efficacy for Behcet’s uveitis patients (142). But related evidence is still inefficient and more studies are needed to better estimate the long-term efficacy and safety of the ocular involvement.

Application of etanercept in Behcet’s uveitis were marginally reported in case reports and small series (143–145). Etanercept may be considered for uveitis in BD patients intolerant to infliximab or adalimumab.

### 3.3 Interleukin blockers

More and more researches have demonstrated the application of bio-agents targeting interleukins and related receptors, including IL-1 blocker, IL-6 blocker, IL-17 blocker, and a monoclonal antibody targeting IL-12/IL-23 (146, 147). A multicenter retrospective observational study, including 19 BD uveitis patients treated with IL-1 blockers anakinra and canakinumab, showed significant effectiveness, remission and decreased steroid dosages in ocular impairment (148). Effectiveness of IL-1 blockers for Behcet’s uveitis was also reported in other small-sample studies (149–151). Tocilizumab is a monoclonal anti-IL-6 antibody. A multicenter study including 11 BD uveitis patients who were refractory to conventional and biologic immunosuppressors showed a rapid and sustained improvement within all optical indicators after the use of tocilizumab (152). Other case reports also illustrated the efficacy of tocilizumab in treating refractory BD uveitis (153, 154). Secukinumab is a human monoclonal anti-IL-17 antibody. It has been reported to inhibit eye inflammation in active non-infectious uveitis patients (155). However, a multicenter RCT including 118 Behcet uveitis patients found no significant effectiveness in the recurrence of uveitis compared with placebo control groups, in which high doses of concomitant immunosuppressive drugs were used in both groups (156). Additional studies are desired to better understand the efficacy and safety of secukinumab in Behcet’s uveitis. Ustekinumab is an inhibitor targeting IL-12/IL-23, and was reported to effectively suppress ocular inflammation in BD patients (157–159).

### 3.4 Treatment with pregnancy

Experts have recommended the use of infliximab, etanercept, and adalimumab in pregnant women, with a FDA category B where reproduction researches in animal models did not observe risk to the fetus and data in pregnant women are not enough or well-established (160). Infliximab, an IgG1 antibody, will transfer the placenta during the second and third trimesters of pregnancy and can be detected in infant’s blood for months after birth (161–164). Evidence from more than 300 pregnancies suggested that infliximab has low fetal risk and is compatible with use within conception and the first two trimesters (165). Consideration should be taken in discontinuation of infliximab early in the third trimester or at the end of the second trimester in order to reduce late fetal exposure to the full extent (165). In addition, in 2020 ACR recommended continuing TNF-α antagonists therapy with infliximab, etanercept, adalimumab, or golimumab in the first two trimesters, and discontinuation should be considered in the third trimester (166–168). However, evidence mentioned above were mainly from Crohn’s disease, rheumatoid arthritis and systemic lupus erythematosus patients. More data about efficacy and safety are needed focusing on BD patients with ocular involvement.

## 4 Conclusion

BD is a chronic multi-systemic disease featured by relapsing-remitting systemic manifestations. Ocular involvements are quite common in BD and may cause severe tissue damage and lead to poor prognosis. Even though the mechanisms of BD remains unclear, advances in genetic and immunological fields have improved our understanding of the immunopathogenesis of BD ocular involvements. Environmental triggers are suggested to be implicated in the pathogenesis of BD in patients with genetic susceptibility. Abnormal autoantigens including HSP60, retinal S-antigen and IRBP might play important parts in ocular BD development via cross reaction. *HLAB51* allele accounts for about 19% of the genetic susceptibility and is the most powerful genetic factor in BD. Accumulating evidence have reported various genetic variants including HLA Related Genes (such as SNPs in *ERAP1* and *MICA* gene) and genes outside the MHC region (such as SNPs in *IL-10* gene, *IL-12/23* gene and others) to be associated with ocular BD. In addition, activation of innate and adaptive immune responses and immunological aberrations were observed. NKT cells, γδT cells, Th cells, Treg cells and cytokines with different functions are assumed to play important roles in the development of BD ocular involvement and might be potential targets for treatment. Novel biotherapies such as IFN-α-2a, TNF-α antagonists and agents targeting interleukins and their receptors have gained increasing attention in recent years. They taget specific molecules in immune response and inflammation process and therefore suppress ocular inflammation in BD. They have shown significant effectiveness, improved remission and prognosis in ocular manifestations of BD. They may make up for the deficiency of conventional immunosuppressors and bring promising prospects for the management of ocular BD. Further studies including RCTs targeting novel bio-agents and mechanisms towards potentially new targets are needed. Finally, the management of BD patients with pregnancy are suggested with infliximab, etanercept, and adalimumab.

## Author contributions

SL and ZX collected related research papers. SL, ZX and JF participated in the manuscript writing for this review. JF and ZL developed research plan and modified the draft. SL and ZX contributed equally in this research. BX gave directive opinions during revision and made great contribution, including revising the manuscript. All authors contributed to the article and approved the submitted version.
